# The effect of contouring instruments on immediate quality and porosity of direct restorations

**DOI:** 10.1007/s00784-025-06342-0

**Published:** 2025-04-22

**Authors:** Carlos Soler-Tornero, Pekka Toivonen, Jaakko Suorsa, Sakari S. Karhula, Simo Saarakkala, Vuokko Anttonen, Jukka Leinonen

**Affiliations:** 1 Institute of Dentistry, School of Medicine, University of Eastern Finland, P.O. Box 1627, Kuopio, FI-70211 Finland; 2 Research Unit of Population Health, Faculty of Medicine, University of Oulu, Oulu, Finland; 3 Department of Oncology and Radiotherapy, Oulu University Hospital, Oulu, Finland; 4 Research Unit of Health Sciences and Technology, Oulu University, Oulu, Finland; 5 Biocenter Oulu, University of Oulu, Oulu, Finland; 6 Department of Diagnostic Radiology, Oulu University Hospital, Oulu, Finland

**Keywords:** Glass ionomer, Micro-computed tomography, Composite resins, Dental materials, Ceramics

## Abstract

**Objective:**

The study aims to evaluate the effect of contouring instruments on the porosity and immediate quality of direct dental restorations.

**Materials and methods:**

Fifteen human molars with 30 Class II and 10 Class V cavities were restored by five voluntary dentists using three contouring instruments (conventional steel, silicone-tipped and diamond-like carbon coated-instruments) and three filling materials (Admira Fusion, Filtek Supreme XTE and Fuji II LC). The restorations were evaluated for immediate quality, porosity and number of pores using stereomicroscope and micro-computed tomography. Statistical analysis included the Shapiro‒Wilk test for normality, one-way ANOVA with Holm‒Sidak post hoc test for normal data, Kruskal‒Wallis ANOVA and Dunn’s test for non-normal data, and Fisher’s exact test for restoration quality comparisons. Statistical significance level was set at *p* < 0.05.

**Results:**

The proportion of restorations with acceptable immediate quality was higher for the restorations that had been contoured using a diamond-coated non-stick contouring instrument compared to the restorations that had been contoured using a conventional steel instrument (*p* = 0.033). The number of pores and porosity were similar for restorations that had been contoured with different contouring instruments. However, the number of pores and porosity were lowest in the restorations made of Filtek Supreme XTE followed by Admira Fusion and Fuji II LC.

**Conclusion:**

The use of diamond-like carbon-coated contouring instruments increased the proportion of acceptable composite restorations compared to conventional steel instruments.

**Clinical relevance:**

Non-stick contouring instruments should be considered for wider use.

## Introduction

Replacement restorations comprise 60% of all direct restorations. The most common reasons for direct restoration repair and replacement are secondary caries and bulk/marginal fractures [1]. Secondary caries lesions are most often found gingivally, whereas bruxism is typically associated with fractures [2, 3].

In addition to patient-related factors, the primary characteristic associated with secondary caries lesions is the presence of biofilm, often associated with a gap at the margin of the restoration [4]. These gaps may result from improper composite filling material compaction or contouring, polymerization shrinkage or insufficient light-curing, which can lead to subsequent degradation of the restoration [4–6]. Also, a rough restoration surface may lead to greater accumulation of organic residue i.e. marginal staining, cause gingival inflammation, or contribute to the development of secondary caries lesions [7, 8]. From a patient’s viewpoint, a rough restoration surface can cause discomfort or irritation to the tongue [9].

Pores can reduce the compressive fatigue limit and strength of composite restorations, and large pores are particularly associated with a higher risk of composite restoration fracture [10, 11]. Additionally, porosity is linked to increased water sorption, which promotes hygroscopic and hydrolytic activities such as swelling [12].

To minimize marginal gaps, roughness, and pores, the handling of filling materials should be optimized. In this context, the stickiness of contouring instruments to filling materials is an important property to consider. The stickiness of the material improves its adaptation to the cavity walls [13]. However, a portion of the filling material may be pulled back from the cavity wall or previous increment if the filling material sticks to the contouring instrument [14]. The stickiness of a basic steel contouring instrument to composite has been determined for several composites [15, 16]; whereas less attention has been given to determining stickiness of different contouring instruments to composite [13, 17]. To the best of our knowledge there are no studies that would have determined the effect of non-stick contouring instruments to the immediate quality or porosity of a direct restoration. Thus, the aim of the present study was to determine the effect of using non-stick contouring instruments on the immediate quality and porosity of direct restorations made from different commercially available materials. The null hypotheses were (1) that the use of non-stick contouring instruments is not associated with the immediate quality, porosity or number of pores of direct restorations (2) that the type of filling material is not associated with the immediate quality, porosity or number of pores of direct restorations.

## Materials and methods

### Sample Preparation

The use of human teeth for this study was approved by the Finnish Student Health Service. Our study does not fulfill any of the criteria that would necessitate obtaining a statement from the Ethics Committee of Human Sciences of Oulu University and thus we did not apply for one. Non-carious human third molars, extracted during routine dental care at the Finnish Student Health Service, were obtained and stored in an antibacterial solution containing 0.9% NaCl and 0.02% NaN_3_ to maintain the teeth’s structural integrity and disinfect them. The teeth were then carefully inspected and selected using an endodontic stereomicroscope, discarding those with defects or abnormalities. Additionally, we measured the teeth to ensure that their size was sufficient for the planned cavity preparations and that the gingival margin of the cavities would be on enamel.

Ultimately, fifteen third molars were selected for this study. The remaining soft tissue remnants were carefully removed, and the teeth were rinsed with water. Two class II cavities were prepared on each tooth. They had an occlusal-apical height of 4 mm, bucco-palatinal depth of 3 mm and mesio-distal width of 3 mm medially and 4 mm approximally. In addition, one class V cavity (height 3 mm, width 5 mm and depth 2 mm) was prepared on ten teeth. The size of the cavities was measured using a periodontal probe and cavity dimensions were further ensured by using specific replicas of class II and class V model cavities. The teeth were then randomly divided and cast in plaster blocks in groups of three. Finally, they were stored in a 0.9% NaCl and 0.02% NaN_3_ solution at + 4 °C until the restorations were made.

### Participants and pre-filling procedures

There were no previous studies on the topic that would have enabled sample size calculations. However, due to recruitment challenges, five voluntary dentists were ultimately enlisted as participants. Three of the participants were undergraduates and two were postgraduates. All Finnish dentists receive comprehensive training in filling therapy during their undergraduate studies. Notably, the two postgraduates in our study had not received additional training in filling therapy. Consequently, we deemed the participants to have equivalent qualifications in filling therapy.

**Fig. 1:**
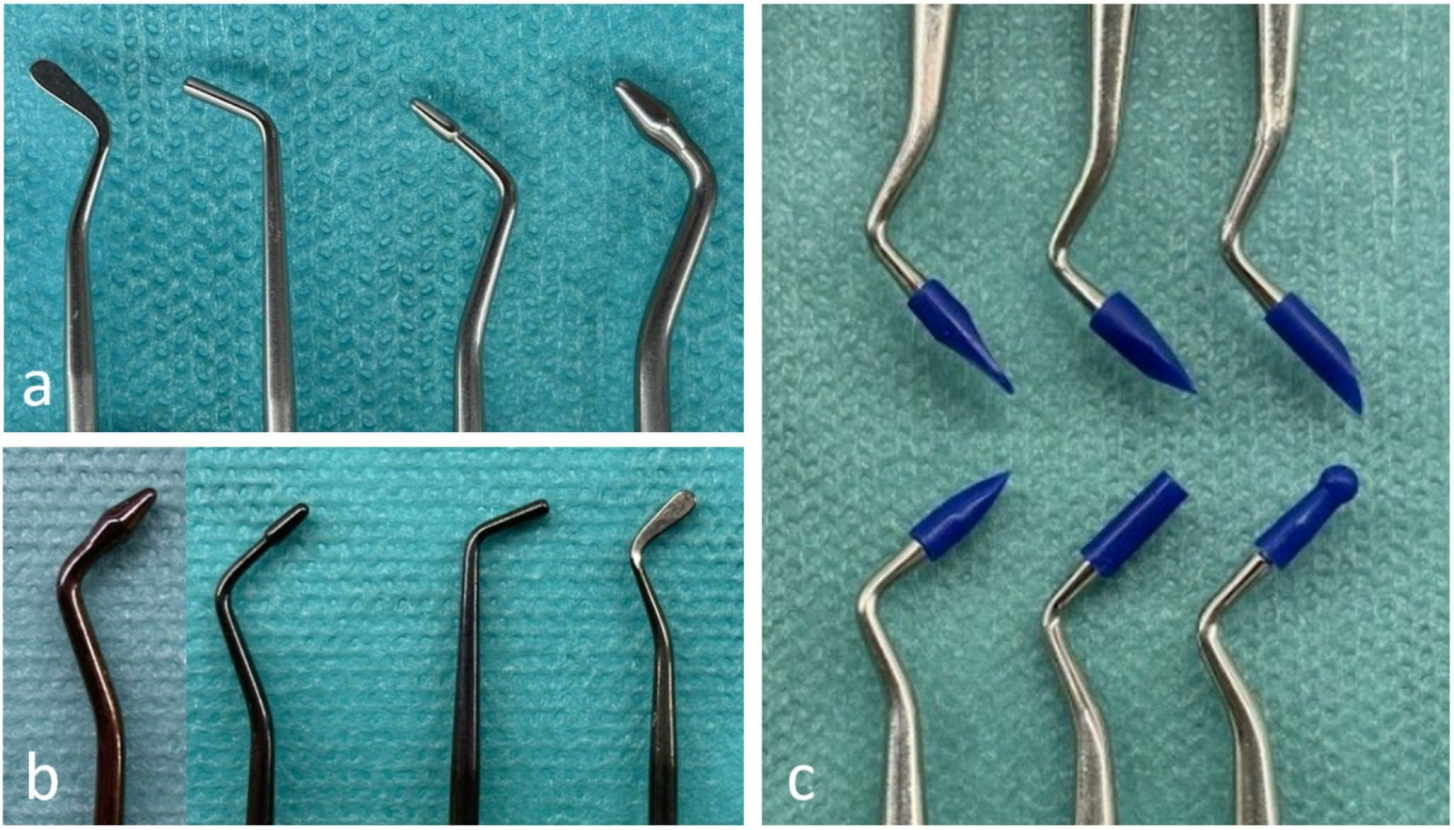
Different types of contouring instruments used during the study: (**a**) Conventional steel instruments (LM-Dental, Parainen, Finland). (**b**) Diamond-like carbon-coated instruments (LM ErgoSense Dark Diamond^®^, LM-Dental, Parainen, Finland). (**c**) Single use silicone-tipped instruments (OptraSculpt^®^, Ivoclar Vivadent, Schaan, Liechtenstein)

First, the participants were instructed to fill the approximal cavities with composite in three increments and then two class V cavities in one increment of glass ionomer. Three different contouring instruments were used: single use silicone-tipped instruments (OptraSculpt^®^, Ivoclar Vivadent, Schaan, Liechtenstein), diamond-like carbon-coated instruments (LM ErgoSense Dark Diamond^®^, LM-Dental, Parainen, Finland) and conventional steel instruments (LM ErgoSense^®^, LM-Dental, Parainen, Finland). The instruments used in the study are illustrated in Fig. 1. The use of silicone-tipped and diamond-like carbon-coated instruments was introduced to the participants before the fillings were made. Just before the volunteer dentists performed the filling procedures, the authors (PT, JS) prepared the teeth for the filling procedures:

1. AutoMatrix (Dentsply Sirona, Konstanz, Germany) bands were tightened around every tooth and wooden wedges were placed approximally.
2. In addition, rubber bands were placed around the teeth in each block to help keep the matrix tight for the cavities at the edges of the blocks.
3. The cavities were total etched with 32% orthophosphoric (Uni-EtchⓇ, Bisco, Anaheim, CA, USA) acid for 15 s.
4. The etchant was rinsed off thoroughly with water for at least 15 s.
5. The cavities were air-dried until slightly moist using a three-way syringe to create a gentle air stream for about five seconds.
6. Scotchbond Universal Adhesive^®^ (3 M ESPE, Seefeld, Germany) was applied in the cavity and rubbed it into the cavity surfaces for 20 s.
7. The evaporation of dilutants in the adhesive was accelerated with a gentle air stream from a three-way syringe for five seconds.
8. The adhesive was light-cured for 20 s at a constant irradiance of 1200 mW/cm² using a Satelec Mini LED^®^ (Acteon, Bordeaux, France) light-curing unit.

### Filling procedure

**Fig. 2:**
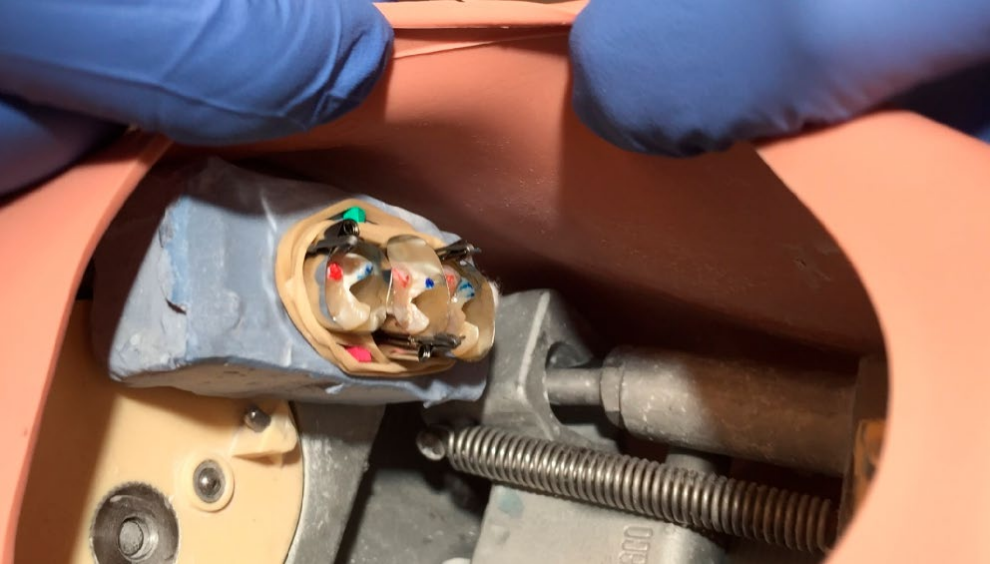
Unrestored tooth specimens after the pre-filling procedures. The plaster block containing the teeth was attached to the left maxillary quadrant of a phantom head

The filling procedures were performed in a dental phantom skull to simulate an actual working environment. The plaster blocks with the teeth in them were attached to the upper left quadrant of the phantom head using double sided tape and glue (Fig. 2). One of the authors (JS or PT) acted as a dental assistant and ensured that the volunteer followed the study protocol. The mesial cavity on each tooth was filled with nanofilled resin-composite (Filtek Supreme XTE^®^, 3 M ESPE, Seefeld, Germany) and distal cavity with a nanohybrid organically modified ceramic (ormocer) based composite (Admira Fusion^®^, Voco, Cuxhaven, Germany). The resin-based composite capsules were disguised so that the volunteers could not know the brand of the resin-based composite they were using. Each volunteer used three different contouring instruments to perform two fillings (one of both resin-based composites). The materials used in the study are listed in Table 1.

**Table 1:** Names, manufacturers and compositions of the materials used in the study

Name	Manufacturer	Composition
Scotchbond Universal Adhesive	3 M ESPE, Seefeld, Germany.	BisGMA (15–25%); HEMA (15–25%); water (10–15%); ethanol (10–15%); silane-treated silica (5–15%); 10-MDP (5–15%); 2-propenoic acid, 2-methyl-, reaction products with 1,10-decanediol and phosphorous oxide (P_2_O_5_) (1–10%); copolymer of acrylic and itaconic acid (1–5%); dimethylamino-benzoat(-4) (< 2%); camphorquinone (< 2%); (dimethylamino) ethyl methacrylate (< 2%); methyl ethyl ketone (< 0.5%); silane (< 1%).
Uni-Etch	Bisco, Anaheim, CA, USA.	32% phosphoric acid; water; synthetic amorphous silica, polyethylene glycol, aluminum oxide.
GC Cavity Conditioner	GC, Tokyo, Japan.	Polyacrylic Acid (20%); Aluminum Chloride Hexahydrate (3%); Water.
Filtek Supreme XTE	3 M ESPE, Seefeld, Germany.	Silane-treated ceramic (60–80%); silane-treated silica (1–10%); UDMA (1–10%); BisEMA (1–10%); BisGMA (1–10%); silane-treated zirconia (1–5%); PEGDMA (< 5%); TEGDMA (< 1%); photoinitiator (trade secret).
Admira Fusion	Voco, Cuxhaven, Germany.	Silicon Oxide (60–80%), glass ceramic fillers (10–20%), ORMOCER resin matrix (10–20%), camphorquinone (< 1%), pigments (< 1%).
Fuji II LC	GC, Tokyo, Japan.	Liquid: Polyacrylic acid (20–22%); HEMA (35–40%); Proprietary Ingredient (5–15%); 2,2,4, Trimethyl hexamethylene dicarbonate (5–7%) TEGDMA (4–6%); photoinitiator (trade secret). Powder: Alumino-fluoro-silicate glass (amorphous) (100%).
Fuji Varnish	GC, Tokyo, Japan.	Isopropyl acetate (40–70%); Acetone (10–30%), cornmint oil (< 1%), cinnamaldehyde (< 1%).

10-MDP: 10-Methacryloyloxydecyl Dihydrogen Phosphate; BisGMA: Bisphenol A diglycidyl ether dimethacrylate; BisEMA: Bisphenol A polyethylene glycol diether dimethacrylate; HEMA − 2-hydroxyethyl methacrylate; PEGDMA: Polyethylene glycol dimethacrylate; TEGDMA: Triethylene glycol dimethacrylate; UDMA: Diurethane dimethacrylate; ORMOCER: Organically Modified Ceramic

We instructed the participants to apply composite resin in oblique increments of no more than 2 mm in height. Each increment was light cured for 20 s using a Satelec Mini LED^®^ (Acteon, Bordeaux, France) light-curing unit at a constant irradiance of 1200 mW/cm². When all six composite resin fillings had been placed the AutoMatrix bands were removed. Then the class V cavities were pre-treated by the authors (JS or PT) as follows:

1. Cavity Conditioner (GC, Tokyo, Japan) was applied and rubbed for 20 s.
2. The Cavity Conditioner was washed off with water for at least 20 s.
3. The cavity was air-dried until slightly moist (after about five seconds) using a three-way syringe to create a gentle air stream.

**Fig. 3:**
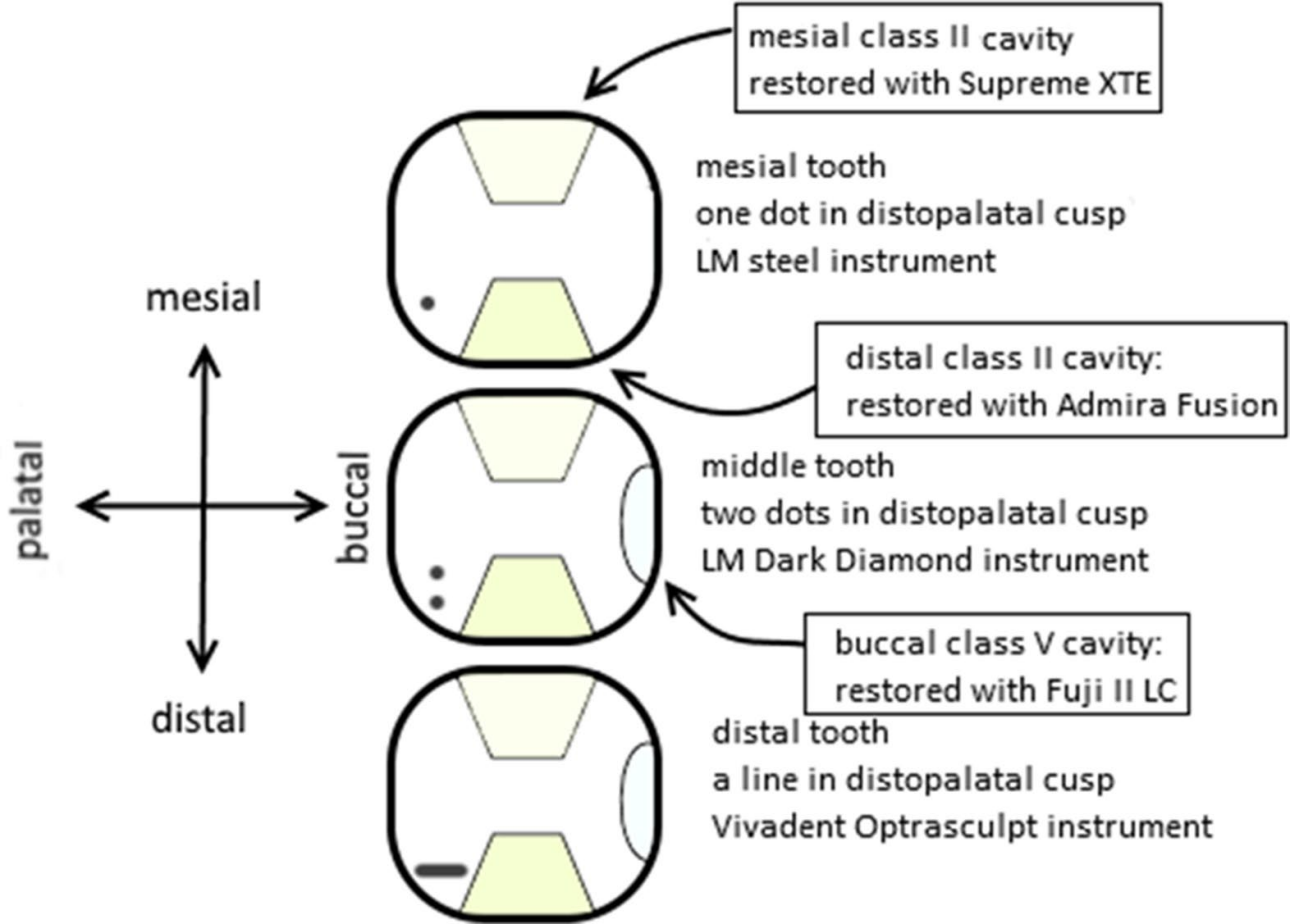
Layout of tooth specimens and restorations. Each plaster block had three teeth, with six class II cavities and two class V cavities. Teeth were identified by marks made with a dental bur on their distopalatal cusps to keep track of the teeth. Each tooth in a block was restored using a different contouring instrument

Subsequently each volunteer dentist filled two class V cavities with light-curable resin-reinforced glass ionomer cement (Fuji II LC^®^, GC, Tokyo, Japan), using OptraSculpt^®^ contouring instrument for the first cavity and for the second cavity a micro-brush moistened with Fuji Varnish^®^ (GC, Tokyo, Japan). The class V cavities were filled using a single increment of 2 mm in depth. Contoured fillings were coated with Fuji Varnish^®^ and light-cured for 20 s at a constant irradiance of 1200 mW/cm² using a Satelec Mini LED^®^ (Acteon, Bordeaux, France) light-curing unit. A diagram of the experimental setup is illustrated in Fig. 3.

Polishing interproximal areas poses considerable challenges, as it can unintentionally cause defects or weaken the proximal contact. To prevent these issues, no polishing or finishing protocol was performed on the restorations during the study. After all the fillings had been placed the dental plaster blocks were crushed cautiously with plaster pliers. The teeth freed from the blocks were immersed in 0.9% NaCl and 0.02% NaN_3_ solution for a minimum of 24 h to ensure full polymerization of the restorative material. Following this initial period, we limited the storage duration in the solution to under one month before micro-computed tomography.

### Micro-computed tomography analysis of the restorations

After a minimum of 24 h following the placement of the restorations, the teeth with the restorations were scanned using micro-computed tomography scanning device (Skyscan 1272^®^, Bruker, Kontich, Belgium). The parameters used for the acquisition were as follows: source voltage 100 kV; current 10 µA; 5 μm pixel; exposure time: 3886 ms; image resolution: 2888 × 2792 pixels; total sections: 2405; rotation step 0.2° and total rotation of 360°, which gave an average scanning time of 7 h and 46 min.

The gaps in the margins of the restorations that extended to the approximal surface were measured for maximum width, length and height using DataViewer software (version 1.5.4.0, Bruker, Kontich, Belgium). The restorations with marginal gaps exceeding 250 μm in all three dimensions and/or with a depth greater than 2 mm were considered unacceptable [9]. Additionally, gaps between composite layers that were 250 μm in all three dimensions and extended to the approximal outer surface of the restoration were considered unacceptable surface roughness. For measuring the porosity and number of pores, the scanned images were evaluated using CT-Analyzer software (version 1.15, Bruker, Kontich, Belgium). The restoration was defined as the region of interest. Then, the threshold was determined based on the density difference between the pores and the restorative material. The amount of porosity was described as the volume percentage of pores per volume unit (vol%).

### Stereomicroscope evaluation of the restorations

The approximal surface and margins of each filling were also evaluated using endodontic stereomicroscope and a dental explorer. Axial and gingival margins and the approximal surface were given a score according to modified United States Public Health Service (USPHS) criteria. Marginal adaptation was assessed as no underfill (0), explorer catches to a small underfill (1), crevice at margin but only enamel exposed (2) and obvious crevice at margin with dentin exposed (3). Surface roughness was assessed as smooth surface (0), slightly pitted surface (1), rough surface (2) or deeply pitted surface (3). Score 0 was considered excellent, scores 1–2 were considered acceptable and score 3 was considered unacceptable. In each restoration, the buccal and palatinal axial margins were considered as one i.e. they were given a single shared score based on the margin that had scored worst. USPHS sum scores were calculated to assess the overall quality of the composite restorations.

### Statistical analysis

Statistical analyses were carried out with the help of the statistical software SigmaPlot (version 15; Systat Software Inc., Palo Alto, CA, USA). Normal distribution of the data was verified with the Shapiro–Wilk test. One-way analysis of variance (ANOVA) and multiple comparisons with Holm–Sidak’s post hoc test were used to assess the statistical significance of normally distributed variables. In cases where the data did not follow a normal distribution, Kruskal–Wallis one-way ANOVA on ranks and Dunn’s test were performed instead. Fisher’s exact test was used to compare the rates of acceptable and unacceptable restorations between operators, materials and instruments. Statistical significance level was set at *p* < 0.05.

## Results

In class V restorations, there were no unacceptable Fuji II LC restorations regardless of the contouring instrument or operator. However, four approximal composite restorations had unacceptable gaps in their margins according to the stereomicroscope assessment. Additionally, two more marginal gaps and one unacceptably rough surface were identified in the composite restorations using micro-computed tomography. The unacceptably rough surface was a gap between composite layers that in fact extended all the way to dentin. Half (5/10) of the composite restorations contoured using the conventional steel instruments were unacceptable. In contrast, only two of the ten composite restorations contoured with the non-stick silicone-tipped instrument were unacceptable. None of the ten composite restorations contoured using the non-stick diamond-like carbon-coated instrument were unacceptable. Fisher’s exact test revealed a statistically significant difference in the proportion of acceptable composite restorations between the restorations contoured using diamond-like carbon-coated instruments and conventional steel instruments (*p* = 0.033). However, the differences in the proportion of acceptable composite restorations between the restorations contoured using silicone-tipped instruments and conventional steel instruments (*p* = 0.350) or diamond-like carbon-coated, and silicone-tipped instruments (*p* = 0.474) were not statistically significant. In addition, there were no statistically significant differences in the proportions of acceptable composite restorations between operators or restorative materials (Table 2).

**Table 2:** Proportion of acceptable restorations according to the composite filling material, contouring instrument and participant. The difference in the proportion of unacceptable restorations was statistically significant between restorations made with conventional steel and diamond-like carbon-coated contouring instruments

Characteristic	Acceptable restorations
Composite filling material	
Supreme XTE	11/15
Admira Fusion	12/15
Contouring instrument	
Conventional steel	5/10
Silicone-tipped	8/10
Diamond-like carbon-coated	10/10
Participant	
1	3/6
2	4/6
3	5/6
4	5/6
5	6/6

**Fig. 4:**
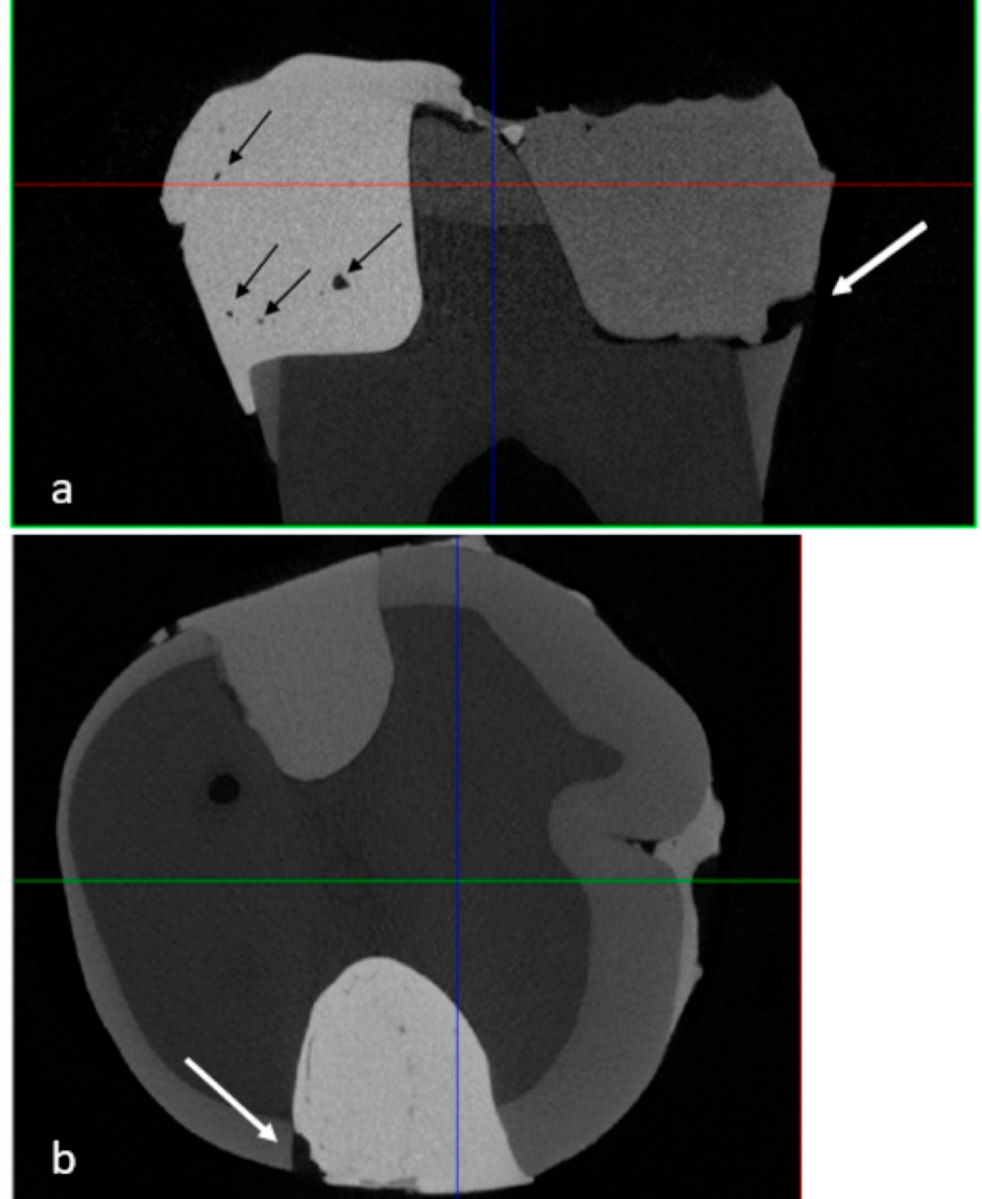
Micro-computed tomography images (**a**) Unacceptable gap at the gingival margin of the right-side restoration made of Filtek Supreme XTE (white arrow). Pores can also be observed inside the left-side restoration made of Admira Fusion (black arrows). (**b**) Unacceptable gap at the axial margin of a restoration made of Admira Fusion (white arrow)

**Fig. 5:**
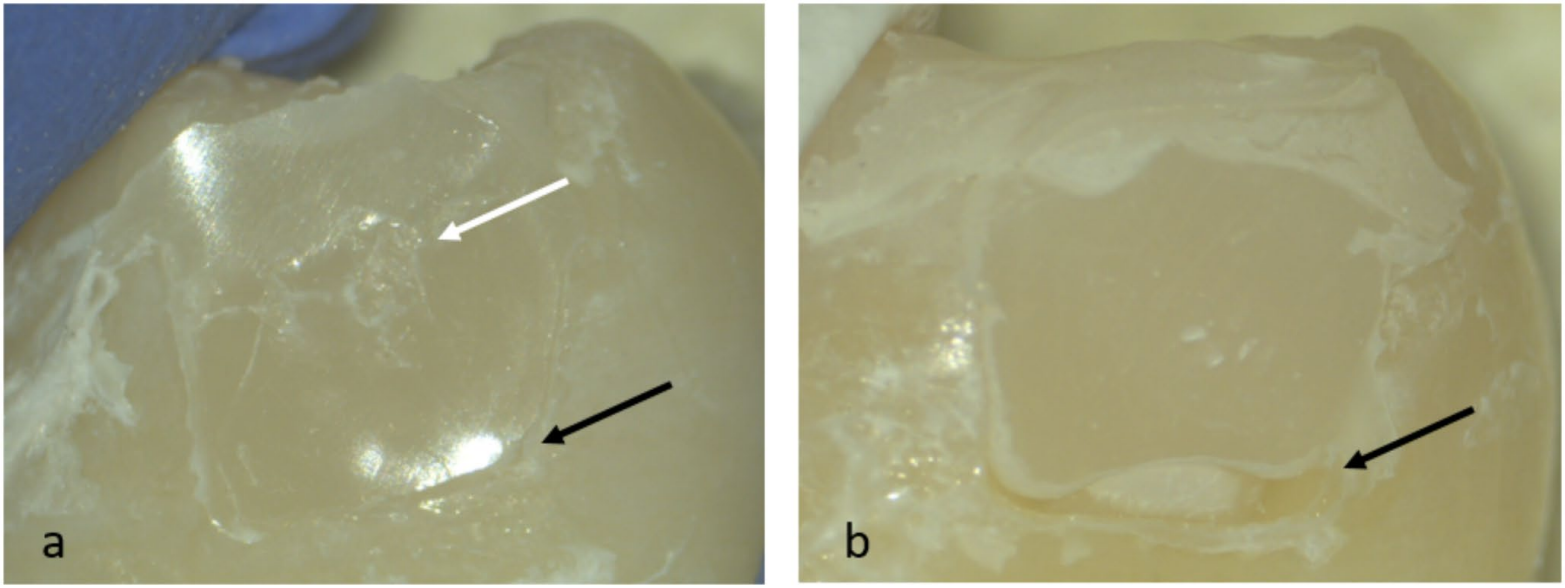
Representative images from the stereomicroscope evaluation: (**a**) An acceptable restoration presenting a small underfill gingivally (black arrow) and some surface roughness (white arrow). (**b**) An unacceptable restoration with a significant crevice at the margin exposing dentin (black arrow)

Three of the gaps were in the gingival margin, two in the axial margin, one in the conjunction of axial and gingival margin and one between composite layers. Representative images from micro-computed tomography and stereomicroscope evaluation are shown in Figs. 4 and 5.

**Fig. 6:**
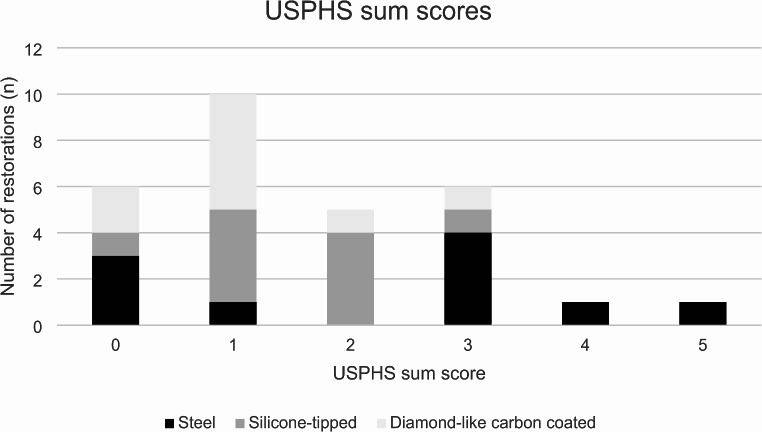
USPHS sum scores for composite restorations contoured with different contouring instruments. The lower the sum score, the better the quality

The results of the one-way ANOVA analysis showed that mean USPHS sum score was 1.1 (SD = 0.9) for the restorations contoured using diamond-like carbon-coated instruments, 1.5 (SD = 0.9) for the restorations contoured using silicone-tipped instruments and 2.2 (SD = 1.8) for the restorations contoured using conventional steel instruments (*p* = 0.162). The distribution of the USPHS scores depending on the contouring instrument used is shown in Fig. 6. The USPHS sum scores were similar between operators (*p* = 0.316) and restorative material (*p* = 0.269).

**Fig. 7:**
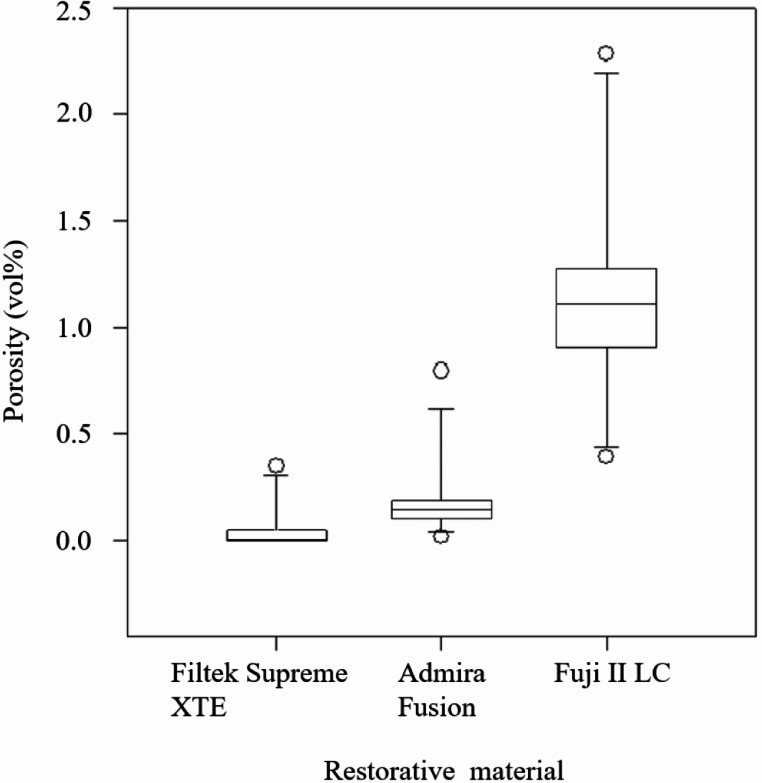
Box plot illustrating the porosity (vol%) in restorations made of different filling materials. Each box represents the interquartile range with the median line inside, and the whiskers show the range of the data excluding outliers, which are depicted as individual points

**Fig. 8:**
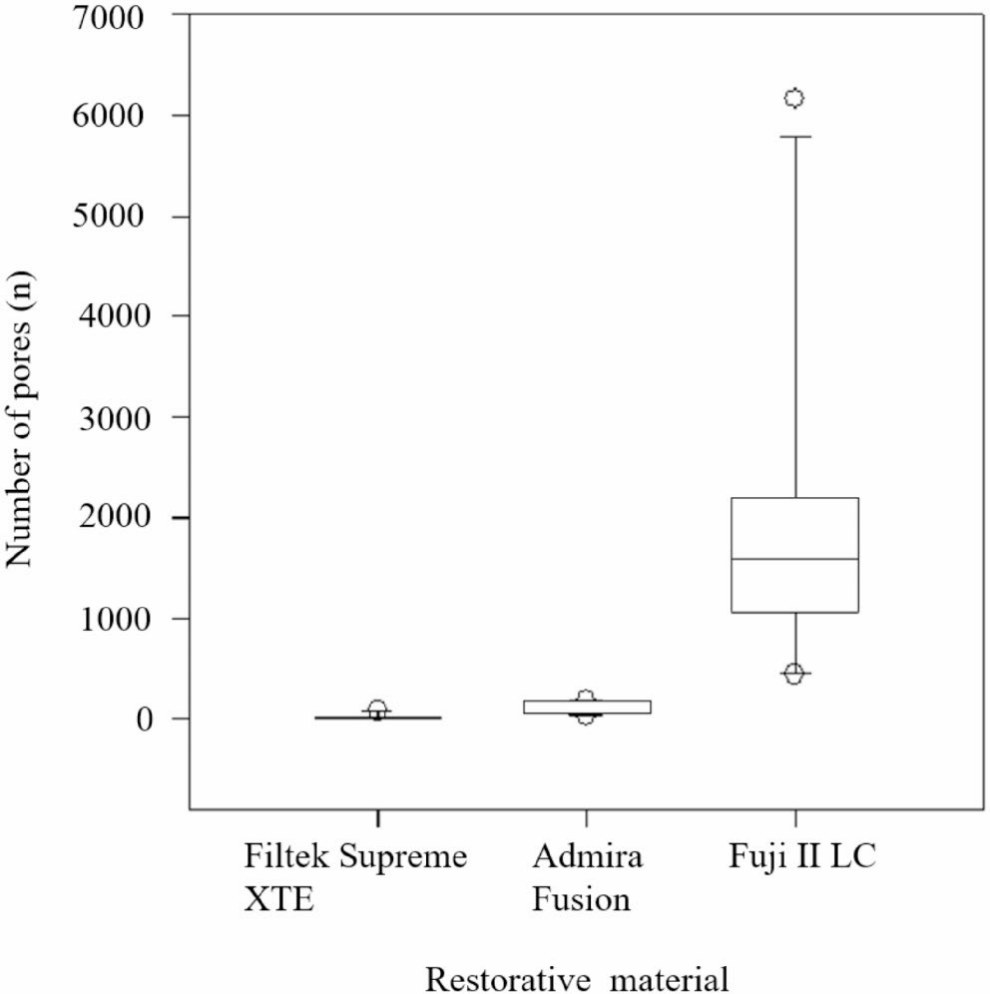
Box plot illustrating the number of pores in restorations made of different filling materials. Each box represents the interquartile range with the median line inside, and the whiskers show the range of the data excluding outliers, which are depicted as individual points

The Kruskal**–**Wallis ANOVA revealed no statistically significant differences in porosity (*p* = 0.468) or the number of pores (*p* = 0.747) between restorations contoured with different instruments. Similarly, no significant differences were found between restorations contoured by different operators for porosity (*p* = 0.954) or pore number (*p* = 0.793). However, the total number of pores and porosity varied according to filling materials (Figs. 7 and 8). The porosity was 0.005% for Filtek Supreme XTE restorations, 0.144% for Admira Fusion restorations, and 1.113% for Fuji II LC restorations. The porosity was significantly lower in the Filtek Supreme XTE restorations compared to the Admira Fusion (*p* = 0.039) and Fuji II LC (*p* < 0.001). The difference between Admira Fusion and Fuji II LC restorations regarding porosity was also statistically significant (*p* = 0.007). Although the Fuji II LC restorations were about 29% smaller than the composite restorations, the Fuji II LC restorations had a significantly higher number of pores (mean = 1579, SD = 1610), compared to Supreme XTE (mean = 4, SD = 26), and Admira Fusion (mean = 61, SD = 55). The difference in number of pores between Fuji II LC restorations and Supreme XTE was statistically significant (*p* < 0.001), as was the difference between Fuji II LC and Admira Fusion (*p* = 0.011). The difference in the number of pores between the Filtek Supreme XTE resin-composite and the Admira Fusion restorations were also statistically significant (*p* = 0.012).

## Discussion

The results of the present study revealed that there are fewer unacceptable composite restorations when the non-stick contouring instruments with diamond-like carbon coating were used instead of the conventional steel instrument. However, the use of the non-stick instruments was not associated with the porosity or the number of pores in the restorations nor the proportion of unacceptable glass-ionomer restorations. Thus, the first null hypothesis “the use of non-stick contouring instruments is not associated with the immediate quality, porosity or number of pores of direct restorations” was partially rejected. However, the USPHS sum scores on the restoration quality were not associated with the contouring instrument used, the composite filling material nor operator.

The most likely reason for the high proportion of these unacceptable restorations may result from the somewhat high stickiness of resin composites to steel [17]. In fact, the stickiness of composite to an adhesive is only slightly higher than to steel [17]. Stickiness to the adhesives and previous composite increments is advantageous for composites to achieve optimal adaptation. Conversely, when the restorative material sticks to contouring instruments, the formation of pores and gaps in and around the restoration increases [16, 18].

Non-stick contouring instruments have been introduced to reduce the sticking of filling materials to contouring instruments [19],. Custom superhydrophobic coating is significantly less sticky than steel. Diamond-like carbon and polytetrafluoroethylene (PTFE) coatings are slightly less sticky compared to steel, but titanium nitride coating does not present any advantages over polished stainless-steel instruments regarding the stickiness to composite [20]. In addition to the type of material/coating, the diameter and geometry of the contouring instrument influences the handling of the composite [13, 17]. In our study, the large diameter of the silicone-tipped contouring instrument may have been a reason for some of the bigger flaws seen in the restorations contoured using it. Nevertheless, the putative effect of contouring instrument geometry on restoration quality was not examined in detail and warrants further studies.

Composites with high filler content are viscous, which hinders their flow into the cavity, potentially leading to marginal defects [21]. However, viscous composites are preferred over flowable composites in many instances because of their better mechanical properties and sculptability [16]. Flowable composites are used in less than 1% of composite restorations made of conventional composites in Oulu, Finland [22]. To reduce protocol heterogeneity our participants were not given the opportunity to use flowable composites. Nonetheless, using a flowable composite liner might reduce the proportion of unacceptable restorations in certain combinations of instruments and materials for some dentists. The occurrence of unacceptable voids is multifactorial, with putative high patient and operator effects in addition to filling materials and instrumentation.

Aside from the clinically unacceptable gaps, our results revealed minor voids in the margins of about two-thirds of the composite restorations. The influence of these small marginal defects remains a subject of debate. While some authors assert that marginal gaps larger than 250 μm are needed for biofilm formation [2], some in situ studies report that 30–60 μm gaps increase secondary caries development [23, 24]. The actual importance of the presence of these marginal gaps is difficult to assess due to the multifactorial nature of secondary caries. For example, microleakage affects caries incidence and progression in vitro but not in situ [23, 25–27]. Furthermore, in vitro and in vivo caries models do not correlate, which reduces the clinical relevance of the in vitro studies [28]. Nevertheless, the minor marginal defects may stain and be misdiagnosed as secondary caries.

The results of the present study showed differences between filling materials regarding porosity and the number of pores. Fuji II LC^®^ presented the highest porosity and number of pores by a large difference, followed by Admira Fusion^®^ and Filtek Supreme XTE^®^ restorations. However, there were no statistically significant differences in the proportion of unacceptable restorations between the two studied composite filling materials. Therefore, the second null hypothesis “the type of filling material is not associated with the immediate quality, porosity and number of pores of direct restorations” was partially rejected.

The porosity of the studied filling materials varied significantly. Fuji II LC restorations had the highest porosity, being 223 times more porous than Filtek Supreme XTE restorations. Whereas Admira Fusion restorations were 29 times more porous compared to Filtek Supreme XTE restorations. Whether the magnitude of porosity between the studied materials effects the clinical performance of the studied materials remains debatable. Mechanically, pores are defects/flaws in the material, as it is a discontinued phase of the material with an e-modulus of zero [29]. Pores in direct composite restorations reduce their compressive fatigue limit and strength and can potentially lead to reduced mechanical and aesthetic properties [10]. Porosity is also linked to increased water sorption, increasing hygroscopic/hydrolytic activities such as swelling, hydrolysis of the material, and degradation of the matrix-filler interface of the resin composite [12]. A large increase (from 1.5 to 3%) in the porosity of a composite restoration reduces the compressive strength and fatigue limit by 30–50% [11, 30]. However, the porosity of the two studied composite filling materials was very low (0.005% for Filtek Supreme XTE and 0.144% for Admira Fusion). Thus, although the porosity of Admira Fusion was 29 times higher than the porosity of Supreme XTE, one can not make assumptions on their physical properties nor longevity based on the difference in their porosity.

Previous studies estimate the porosity in composites falling within the range of 0.5–4% of the total volume [31]. There are pores already in the composite filling material that has not been extruded from the compule [32]. Therefore, contouring is not alone responsible for the porosity of the composite. In addition, the findings of the present work showed that different operators were not associated with the proportion of acceptable restorations. The overall low porosity of the composite filling materials in our data may be explained by the use of micro-computed tomography to detect it. Traditionally, porosity has been assessed from sectioned restorations. This technique is only accurate under certain conditions, e.g., very thin sample sections and homogeneity of porosity within the sample [33]. Whereas the micro-computed tomography used in the present study enables precise visualization of the reconstructed structure at high resolution in three dimensions, facilitating effective quantification of voids, porosity and number of pores, which is a strength for the study [10, 18]. The porosity of glass ionomer cements ranges from 0.15 to 4.4% [34, 35]. The variation of porosity in glass ionomer fillings is largely attributed to factors such as composition of the material, operator effect, and the mixing and application techniques used [35]. However, the detection method and threshold may also affect the detected porosity [34].

The major strengths of the study are the use of three different contouring instruments and three restorative materials in an environment that simulated closely actual clinical work. Although reducing the stickiness of the composites to the contouring instruments seems interesting from a logical point of view, scientific literature on non-stick instruments is scarce. This scarcity adds value to our study. Furthermore, our study protocol mimicked clinical settings to a great degree to simulate real-life patient encounters, where succeeding in filling therapy is far from granted. The limitations of the study are the relatively small sample size that resulted from the surprising difficulties in recruiting clinicians to perform the restorations, and the limited prior familiarity of the operators with the non-stick instruments used. In particular, the large tip size of the silicone-tipped instruments may have come as a surprise for our participants. The previous experience of the clinicians with the filling materials used in the study, even if common in general practice, is unknown and could have been also a source of variation. We included different operators in the study, believing it would be more representative of the general dental community. The variation in the proportion of unacceptable restorations among our participants was within the range previously reported [22]. However, we recognize that using a single operator also has advantages, such as reducing variability associated with different techniques and skill levels. Additionally, in this study, we exclusively used visuo-tactile inspection and micro-computed tomography scanning to study marginal adaptation. This choice was driven by our interest in measuring the pores and voids in micrometers in 3D and in accordance with FDI criteria for evaluating dental restorations [9]. Nonetheless, combining scanning electron microscopy and dye penetration techniques with micro-computed tomography could offer advantages in future studies.

To promote understanding of the effect of non-stick contouring instruments on restoration quality and longevity, further studies are needed, including variables such as the time spent contouring the restoration and a larger sample of dentists who are routinized with the use of the contouring instruments and filling materials in the study. A randomized controlled clinical trial would be preferable. However, considering the low failure rate of composite fillings in randomized controlled trials, and the putatively small effect of non-stick contouring instruments alone on the failure rate, a clinical study on the topic might be difficult and arduous to perform.

## Conclusions

The use of diamond-like carbon-coated contouring instruments increased the proportion of acceptable composite restorations compared to conventional steel instruments. The porosity and number of pores in resin-reinforced glass ionomer restorations was significantly higher than the porosity of resin-composite restorations. Furthermore, the Supreme XTE composite restorations had significantly less pores and porosity than the Admira Fusion composite restorations.

## Data Availability

No datasets were generated or analysed during the current study.
